# Manila duck (*Cairina moschata*) frozen semen quality in lactated ringer’s egg yolk-astaxanthin with different concentrations of DMSO

**DOI:** 10.1590/1984-3143-AR2023-0015

**Published:** 2024-12-02

**Authors:** Sipora Petronela Telnoni, Hory Iramaya Dilak, Iis Arifiantini, Wilmientje Marlene Nalley

**Affiliations:** 1 Universitas San Pedro, Faculty of Mathematics and Natural Science, Major of Biology, Kupang, East Nusa Tenggara, Indonesia; 2 Bogor Agricultural University, School of Veterinary and Biomedical Medicine, Major of Reproductive Biology, Bogor, West Java, Indonesia; 3 Universitas Nusa Cendana, Faculty of Animal Science, Major of Animal Science, Kupang, East Nusa Tenggara, Indonesia

**Keywords:** Manila duck, frozen semen, cryopreservation, astaxanthin, DMSO

## Abstract

This study was conducted to evaluate manila duck’s (*Cairina moschata*) frozen semen quality after cryopreservation in lactated ringer's egg yolk-astaxanthin (LREY-A) with 5 different concentrations of dimethyl sulfoxide (DMSO). Methodology: Semen was collected from 3 manila ducks (*Cairina moschata*) using the cloaca massage technique twice a week. Fresh semen was evaluated macro and microscopically then polled and divided into 5 tubes of treatments. Each tube was diluted in DMSO4, DMSO6, DMSO8, DMSO10, and DMSO12. The semen of each treatment was loaded into a 0.25 mL straw and equilibrated at 5 °C for 2 h. Freeze above nitrogen vapor and stored a container of liquid nitrogen at -196 °C, then semen thawed in a water bath at 37 °C for 30 sec. Data were analyzed using One-Way ANOVA Analysis. Results of this showed that post-equilibration sperm motility and sperm viability have differed significantly (P<0.05) for each treatment, with the highest % sperm motility DMSO8 and DMSO6, this is also shown in post-thawing sperm motility and viability which have differed significantly (P<0.05) and the highest % sperm viability were DMSO8 and DMSO6. In conclusion, Frozen semen extender formulation of DMSO8 and DMSO6 which are used in manila duck semen cryopreservation was the best to other treatments to maintain % sperm motility and % sperm viability in post-equilibration and post-thawing. The highest sperm motility recovery rate was in DMSO8. The lowest sperm live and dead abnormality was in DMSO8^.^ It is concluded that the combination of DMSO8 was the best in maintaining the quality of manila duck frozen semen.

## Introduction

The manila duck (*Cairina moschata*) is a species of duck developed in several regions of Indonesia, including East Nusa Tenggara (ENT). Manila ducks in ENT can contribute resources to the genetic diversity of poultry and animal protein food. The intended contribution relates to the preservation and development of manila ducks. Preservation and development of manila ducks can be done through reproductive biotechnology, namely semen cryopreservation. Semen cryopreservation functions to store genetic material for a long time and supports in situ conservation used in livestock selection programs and supports the implementation of artificial insemination (AI) programs. Semen cryopreservation and AI play an important role in preserving and transferring rare genetic material, spreading endangered species, improvement of livestock genetics and have been applied to ducks (Penfold et al., 2001; Sontakke et al., 2004; King et al., 2022).

The success of duck semen cryopreservation is influenced by the quality of frozen semen after thawing and the fertility value of sperm after AI. The quality of frozen duck semen in cryopreservation is based on motility, viability, recovery rate, and fertility of sperm after thawing and insemination of frozen semen. The successful cryopreservation of duck semen currently shows sperm motility of 41.91 percentage (%) in Ringer lactate-glucose with dimethylformamide (DMF) cryoprotectant (Taşkın et al., 2020). The quality of frozen semen in the form of low sperm motility is caused by basic diluents and antioxidants as well as cryoprotectants and cryoprotectant concentrations in duck semen cryopreservation so that frozen semen diluent formulations are needed which contain basic diluents, antioxidants, cryoprotectants, and optimal cryoprotectant concentrations.

Lactated ringer's egg yolk diluent (LREY) is a diluent that has been widely used for the preservation and cryopreservation of poultry semen which aims to maintain the integrity of poultry sperm cells. The use of LREY in local chicken semen cryopreservation was able to maintain sperm motility after thawing and recovery rates with values of 40.83 (%) and 46.71 (%) (Telnoni et al., 2017). Astaxanthin is a good antioxidant and has hydroxyl and ketone groups which play an important role in neutralizing reactive oxygen species (ROS) in cell membranes including sperm cells. (Dai et al., 2020; Brotosudarmo et al., 2020). Astaxanthin 0.004 (%) added to free-range chicken semen was able to maintain sperm motility at 41.03 (%) for 36 hours of storage (Pratiwi et al., 2019).

Dimethylsulfoxide (DMSO) is a cryoprotectant that is often used in poultry semen cryopreservation (Herrera et al., 2005). DMSO has the ability to protect sperm through intracellular water transfer thereby minimizing the formation of ice crystals and reducing salt concentrations (Kulaksiz et al., 2013). DMSO concentration in semen cryopreservation is important for the success of semen cryopreservation. Optimum levels of cryoprotectants depend on the types and different species concentrations of cryoprotectants (CPAs) (Han et al., 2005; Soni et al., 2019).

This research of manila duck semen cryopreservation using diluents lactated ringer's egg yolk-astaxanthin(LREY-A) in 5 different DMSO concentrations 4 (%), 6 (%), 8 (%), 10 (%), and 12 (%) aims to obtain formulations of frozen semen diluents for cryopreservation of manila duck semen in the aim of genetic preservation and population increase for protein food source through AI.

## Methods

### Time and location of study

This study was conducted in October 2022 at the Indonesian Livestock Research Institute (Balitnak), Ciawi-Bogor.

### Approval of research committee

This research was approved by the research implementing committee based on Number: 623/HM.240 issued by the Agricultural Research and Development Agency, Indonesian Livestock Research Institute.

### Experimental animals

The study material was 3 male manila ducks aged ±1 years as a source of semen. Manila ducks were reared in battery cages; each cage was equipped with a feed place and a drinking place. manila ducks are fed a commercial diet with 18 (%) of crude protein). Feed is given 2 times in 1 day i.e. in the morning and evening, and water was given *ad libitum*.

### Extender preparation

The composition and constituents of LREY are 80.0 mL Ringer's lactate, 20.0 mL Egg yolk, and pH 6.8. Formulation of frozen Manila duck semen diluent in 5 treatments with LREY ratios of 96 mL, 94 mL, 92 mL, 90 mL, and 88 mL. For each treatment, 0.004 grams of Astaxanthin and DMSO were added with 5 concentrations, namely 4%, 6%, 8%, 10%, and 12%. furthermore, to each of the 5 treatments, 1000 IU mL-1 Penicillin and 1 mg mL-1 Streptomycin were added and homogenized. LREY was measured and homogenized using a magnetic stirrer for 10 minutes, centrifuging at 3 000 x g for 15 minutes, and the pH was adjusted using Tris aminomethane to pH 6.8. Astaxanthin for each treatment was weighed using digital scales. DMSO in 5 different concentrations was measured using a micropipette.

### Semen collection and evaluation

Manila duck (*Cairina moschata*) semen was collected twice a week by cloaca massage technique (Tan, 1980) in the morning dan the semen was transferred to the laboratory of animal reproductive. Semen was evaluated macro and microscopically. Macroscopic evaluation of semen was carried out for volume, pH, color, and consistency. Semen volume (mL) was measured using a graduated pipette, pH was measured using indicator paper (Merck scale 6.4-8), semen color and consistency were evaluated visually. Microscopic evaluation of semen was carried out to determine mass movement, sperm motility, sperm viability, sperm morphology, and sperm concentration. The movement of the sperm mass was evaluated under a light microscope with 100x magnification. Sperm motility was evaluated under a light microscope at 400x magnification. Sperm viability and morphology were evaluated using the Eosin-Nigrosin staining method according to Blom (1950) with a light microscope with 400x magnification. Sperm concentration was measured using a counting Neubauer chamber or hemacytometer with a light microscope at 400× magnification.

### Semen processing and freezing

Manila duck (*Cairina moschata*) semen demonstrated >70 (%) sperm motility, <20 (%) abnormality, and >3000×10^6^ sperm cells mL^-1^ in the concentration used for this study. To avoid an individual variation, all semen was pooled into 1 tube. Semen diluted in 5 treatments were LREY-Astaxanthin with DMSO 4% (DMSO4), LREY-Astaxanthin with DMSO 6% (DMSO6), LREY-Astaxanthin with DMSO 8% (DMSO8), LREY-Astaxanthin with DMSO 10% (DMSO10), and LREY-Astaxanthin with DMSO 12% (DMSO12). Diluted semen containing 400×10^6^ sperm cells mL^-1^ (100×10^6^ sperm cells straw^-1^), then was packed into mini straws (0.25 mL) and labeled, all straws were placed in a freezing rack and equilibrated at 5 °C for 2 h and were evaluated for their sperm motility (%) and sperm viability (%). Immediately after equilibration, the straws freeze above liquid nitrogen vapor for 10 min and then are immersed into the liquid nitrogen container (-196 °C) for further evaluation after 24 hours.

### Semen analysis after freezing

Straws of semen by 5 treatments were thawed in a water bath at 37 °C for 30 sec. Semen was evaluated for its sperm motility (%) and sperm viability (%). Further, the sperm motility recovery rate (%) was measured by dividing frozen-thawed sperm motility with sperm motility of fresh semen at ×100 (%).

### Statistical analysis

All quantitative data are presented as mean values ± Standard error of the mean (SEM). Data analysis was performed using One-Way ANOVA and compared with Duncan’s multiple range tests if the F-value was significant (P<0.05) in IBM SPSS Statistics Version 2020 package program for Windows.

## Results

Fresh semen characteristics of manila duck are presented in Table 1, fresh semen was evaluated macro and microscopically. The quality of frozen-thawed semen in (%) sperm motility and (%) sperm viability after the cryopreservation of manila duck semen is shown in Table 2 and Table 3. % sperm motility post diluent in each treatment have not differed significantly (P>0.05). The percentage of sperm motility post-equilibration and post-thawing have differed significantly (P<0.05). Formulation of DMSO4, DMSO6, and DMSO8 was higher (p<0.05) than DMSO10, and DMSO12 post-equilibration, the motility decreased significantly when the concentration of DMSO in the LREY-A increased to 10 (%) and 12 (%) in the post-equilibration condition. The percentage of sperm motility in the formulation of DMSO8 and DMSO6 was the highest and differed significantly (P<0.05) from DMSO10, and DMSO12 post-thawing. The lowest of percentage sperm motility of DMSO10 and DMSO12 is influenced by the value of sperm motility post-equilibration.

**Table 1:** Fresh semen characteristics of manila duck based on macro and microscopically.

**Parameters**	**Mean±SEM**
**Color**	Milky White
**pH**	6.51±0.08
**Consistency**	Thick
**Volume (mL)**	0.45±0.07
**Mass activity**	++
**Motility (%)**	75.83±2.36
**Viability (%)**	82.99±1.60
**Abnormality (%)**	2.41±1.29
**Concentration (x 10^6^)**	3733±97.18

SEM: Standard error of the mean.

**Table 2:** Sperm motility of frozen-thawed manila duck semen in RLEY-A extender using 4, 6, 8, 10, and 12% concentrations of DMSO.

**Cryoprotectant** **% DMSO**	**Post dilution**	**Post equilibration**	**Post thawing**	**Recovery rate**
4	66.46±1.59	53.91±3.53^b^	24.02±1.19^b^	32.42±1.05^b^
6	69.61±2.92	60.31±3.64^b^	27.36±1.23^c^	36.78±1.73^bc^
8	69.98±3.22	61.83±1.21^b^	29.43±2.30^c^	38.89±3.00^c^
10	68.00±2.48	40.97±1.88^a^	19.23±0.88^a^	26.50±1.60^a^
12	62.95±3.15	40.26±1.79^a^	17.36±0.65^a^	22.98±1.47^a^

The same columns with different superscripts are differ significantly (p<0.05).

**Table 3:** Sperm viability of Frozen-thawed manila duck semen in RLEY-A extender using 4, 6, 8, 10, and 12% concentrations of DMSO.

**Cryoprotectant** **% DMSO**	**Post dilution**	**Post equilibration**	**Post thawing**
4	72.95±0.59^ab^	56.57±1.19^c^	30.71±0.69^b^
6	74.02±0.52^b^	65.42±1.19^d^	34.14±0.43^c^
8	74.40±0.43^b^	66.80±1.20^d^	35.16±0.18^c^
10	72.23±0.57^a^	52.10±0.59^b^	22.24±0.68^a^
12	71.51±0.48^a^	44.83±1.29^a^	21.62±0.46^a^

The same columns with different superscripts differ significantly (p<0.05).

The percentage of sperm viability of manila duck frozen-thawed semen is shown in Table 3 on post-dilution, post-equilibration, and post-thawing differ significantly (P<0.05) in each treatment. The highest sperm viability post-dilution was shown on DMSO8 and DMSO6, post-equilibration of frozen-thawed semen on DMSO8 and DMSO6 was the highest, and significantly difference (P<0.05) to DMSO4, DMSO10, and DMSO12 with the lowest % sperm viability. Post-thawing sperm viability on DMSO8 and DMSO6 was higher (p<0.05) than DMSO4, DMSO10, and DMSO12.

The percentage of recovery rate in sperm motility of manila duck frozen-thawed semen shown in Table 2, differs significantly (P<0.05) in each treatment. The combination of DMSO12 and DMSO10 has a lower percentage of sperm motility in recovery rate than DMSO4 and DMSO6, but DMSO8 was the highest.

**Figure 1:**
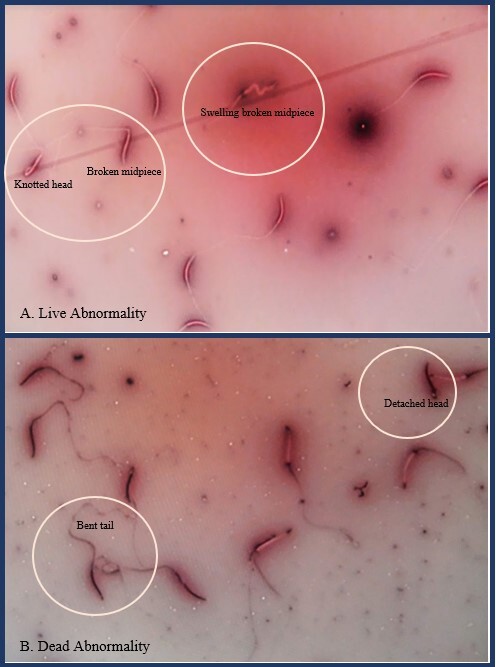
A. Live abnormality in knotted head, broken midpiece, and swelling broken midpiece; B. Dead abnormality in bent tail and detached head under a light microscope at 400× magnification.

The shape of live and dead manila duck sperm abnormality is shown in Figures 1A and 1B. Furthermore, the percentage of live and dead sperm abnormality of manila duck in 3 stages of semen is shown in Table 4. The live and dead sperm abnormality in post-dilution and post-equilibration by means of light microscope evaluation have not differed significantly (p>0.05). Post thawing manila duck sperm live and dead abnormality have differed significantly (p<0.05). The highest percentage of live abnormality was found in DMSO12 and the lowest was found in DMSO8. The highest percentage of dead abnormality was found in DMSO12 and the lowest was found in DMSO8.

**Table 4:** Manila duck sperm abnormality in RLEY-A with different concentrations of DMSO.

**Cryoprotectant% DMSO**	**Sperm Abnormality**	**Post dilution**	**Post equilibration**	**Post thawing**
4	Live Abnormality	1.09±0.18^a^	1.19±0.26^a^	3.81±0.22^bc^
	Dead Abnormality	1.25±0.26^a^	2.23±0.31^a^	3.42±0.19^ab^
6	Live Abnormality	0.90±0.24^a^	1.51±0.28^a^	3.08±0.18^ab^
	Dead Abnormality	1.33±0.65^a^	1.75±0.46^a^	3.60±0.56^ab^
8	Live Abnormality	0.69±0.21^a^	1.80±0.54^a^	2.49±0.14^a^
	Dead Abnormality	1.06±0.31^a^	2.04±0.19^a^	2.83±0.39^a^
10	Live Abnormality	1.70±0.28^a^	2.03±0.58^a^	3.48±0.59^abc^
	Dead Abnormality	0.72±0.15^a^	2.62±0.57^a^	5.37±0.73^bc^
12	Live Abnormality	1.47±0.49^a^	2.21±0.31^a^	4.46±0.45^c^
	Dead Abnormality	1.66±0.54^a^	3.51±0.90^a^	6.87±1.14^c^

The same columns with different superscripts differ significantly (P<0.05).

## Discussion

This study result has similarities to the result of the White Pekin duck, Kuttanad duck, and Khaki Campbell ducks with Orpington Fauve (Kho-01) and Miniducks (K2) (Cyriac et al., 2013; Zawadzka et al., 2015). This shows that the male manila ducks during the preparation for the source of semen are in ideal environmental conditions for raising drakes. Environment plays a crucial role in semen quality and male fertility. Adverse environmental factors can result in poor semen quality with decreased sperm concentration, sperm motility, viability, and normal morphological forms (Kumar and Singh, 2022). Another result of individuals raising Muscovy drake sperm was lower than this study in sperm motility was ±65,57 (%) and sperm viability was ±54,95 (%) (Chen et al., 2016). This lower yield could be due to differences in area factors such as climate and raising management. Semen quality is affected by several factors of which season is one of the major factors. It exerts its effect on reproductive performance through macro and micro climatic factors like temperature, humidity, rainfall, and photo-period (Bhakat et al., 2010) and poultry raising management conditions (Silyukova et al., 2022).

Manila duck frozen semen quality after cryopreservation resulted in a gradual decrease in motility and viability values under conditions of post-equilibration and post-thawing. Equilibration as a stage of adjustment of sperm and diluents including cryoprotectants has an influence in determining the stages of freezing. Equilibration as the total period of sperm contact with a cryoprotectant prior to freezing helps keep sperm membrane integrity as well as their survival (Leite et al., 2010). The highest decrease in sperm motility after post-equilibration was >20 (%), present in the combination of DMSO10, and DMSO12. This shows a negative effect of the high concentration of DMSO in the diluent on the sperm motility of manila ducks. This is related to the toxic effect of cryoprotectants such as DMSO which can affect the stability of cell membranes after being diluted and stored for short several time. The DMSO is toxic to cells at high concentrations and temperatures could be due to its ability to induce non-lamellar structures in phospholipids and enhance membrane permeability that might compromise sperm structures and functions (Whaley et al., 2021; Sum and Pablo, 2003). Furthermore, DMSO8, DMSO6, and DMSO4 were the best levels in combination with lactated ringer egg yolk and astaxanthin for maintaining sperm motility post-equilibration.

Post-thawing is the stage where the sperm is thawed again after being frozen at the freezing point (-196 °C) to 37 °C for 30 sec. At this stage, sperm will experience a temperature transition period due to thermal shock and cryoinjury which affect sperm motility. Thermal shock is an issue when cryopreserving avian sperm, which is during the final stages of differentiation, spermatozoa lose an extensive part of their defensive antioxidants by losing part of their cytoplasm (Najafi et al., 2022; Mehdipour et al., 2020; Zhandi et al., 2020). Cryoinjury is not only limited to the freezing process but it may also occur during the thawing process as the ice melts or recrystallizes (Said et al., 2010) and subsequently with the formation of intracellular and extracellular ice crystals, cellular dehydration, and osmotic shock (Oberoi et al., 2014). The best post-thawing motility values found in the combination of DMSO8 and DMSO6 were 29.43±2.30 (%) and 27.36±1.23 (%), these motility values were similar to those obtained by Svoradová et al. (2018) was 26.66±3.15 (%) on semen cryopreservation of oravka chickens. In addition, the motility values found have not been able to reach the optimum value for AI applications, namely >40%. This is caused by the activity of reactive oxygen species (ROS) in sperm during cryopreservation. The value of motility after thawing has not been achieved due to the negative effects of reactive oxygen species on sperm cells during cryopreservation. During the cryopreservation process, oxidative damages caused by the generation of supraphysiological levels of ROS in sperm could impair cellular functions and survival (Wu et al., 2021).

The best sperm viability value also found in the combination of DMSO8 and DMSO6 was 35.16±0.18 (%) and 34.14±0.43 (%), this is influenced by sperm motility value positively correlated with viability. Sperm motility positively correlates with viability and trends towards a positive association with plasma membrane integrity (Chen et al., 2016). The low viability value in frozen sperm is due to the condition of the avian sperm membrane which has a high sensitivity membrane regarding lipid content during the cryopreservation process. The sperm membrane in avians is a complex and sensitive lipid biomembrane structure that contains high levels of polyunsaturated fatty acids (PUFAs), a highly sensitive to the freeze-thaw process and the deleterious effects of lipid peroxidation in cryopreservation (Long, 2006). A major cause of increased membrane permeability, decreased fluidity, and, more generally, changes in the plasma membrane composition (Surai, 2002; Blesbois, 2011).

The percentage of sperm motility recovery rate of manila duck sperm after thawing showed a combination of DMSO8 was 38.89±3.00 (%) and better than other LREY-A combinations. This is related to the value of post-thawing manila duck frozen sperm motility where DMSO8 gradually is good. This result indicates the ability to restore the sperm cell membrane and its relationship to the ability to use ATP as an energy source. Sperm motility recovery appears to be related to a more rapid and complete recovery of membrane integrity and permeability and perhaps to a more efficient preservation of the biosynthesis and use of adenosine triphosphate (ATP) in the axoneme (Calamera et al., 2010). The values obtained were almost similar to the results for sperm motility recovery rate of Indonesian native chicken semen in the combination of Ringer's lactate egg yolk + DMSO 7 (%) was 35.74±2.00 (%) (Khaeruddin et al., 2020).

Sperm morphology in abnormality is an important part of the implementation for aves semen cryopreservation, which refers to the condition and ability of the sperm in the fertilization process. The morphologic structure of the sperm is extremely important for the processes of fertilization and embryo development (Ozkavukcu et al., 2008). Live and dead abnormality of manila duck sperm on cryopreservation (Figure 1) was an abnormality shape in the head, midpiece, and tail. Live and dead abnormalities of sperm morphology were subdivided into three groups: head, midpiece, and tail defects (Yurchuk et al., 2021). Abnormal shapes of live and dead manila duck sperm (Figure 1A, B) were broken midpiece, knotted head, and swelling broken midpiece; detached head and bent tail. The shape of abnormalities obtained is by the research of Feyisa et al. (2018) in sperm abnormality of four Korean native chicken breeds. The abnormal condition is caused by the condition of osmotic pressure against cold shock during the cryopreservation process, especially after thawing. Sperm tail coiling could be displayed post-thaw due to osmotic challenges (Raad et al., 2017; Gómez-Torres et al., 2017). Midpiece injuries or broken such as bent neck was the most prevalent abnormality after frozen-thawed semen (Váradi et al., 2013; Lukaszewicz, 2002). The head injuries were knotted and detached in the thawed sperm quality of Common Pheasant (Castillo et al., 2021).

Furthermore, the live and dead abnormalities that occurred after dilution and equilibration were the same in all combinations of LREY-A with DMSO4, DMSO6, DMSO8, DMSO10, and DMSO12 in Table 4. This shows all combinations of LREY-A in ^DMSO^ at this stage it can maintain the morphological quality of both live and dead sperm. The abnormality conditions of live and dead sperm at the thawing stage showed significant differences (P<0.05), the lowest live and dead abnormalities found in DMSO8 were 2.49±0.14 (%) and 2.83±0.39 (%). The highest live and dead abnormalities found in DMSO12 were 4.46±0.45 (%) and 6.87±1.14 (%). The % value of this abnormality correlates with the % motility of sperm, where the ability of sperm motility is influenced by the sperm’s morphology. The condition of sperm surviving with abnormalities in the head and tail can reduce the motility rate. The increasing of sperm head and coiling of the sperm tail will impair sperm motility (Pereira et al., 2017). Furthermore, changes in temperature and osmotic pressure affect the decrease in post-thawed motility, with a unique role in Na, K-ATPase (NKA) activity, in response to bending the tail of chicken sperm (Sushadi et al., 2023).

## Conclusions

Frozen semen extender formulation of DMSO8 and DMSO6 which are used in Manila duck semen cryopreservation was the best to other treatments to maintain the percentage of sperm motility and percentage of sperm viability in post-equilibration and post-thawing, whereas all treatments have similar ability to maintain sperm motility and sperm viability post-dilution. Furthermore, the sperm motility recovery rate and live dead abnormality of frozen semen quality in DMSO8 were the best.
